# The roles of impact and inertia in the failure of a shoelace knot

**DOI:** 10.1098/rspa.2016.0770

**Published:** 2017-04-12

**Authors:** Christopher A. Daily-Diamond, Christine E. Gregg, Oliver M. O'Reilly

**Affiliations:** Department of Mechanical Engineering, University of California at Berkeley, Berkeley, CA 94720-1740, USA

**Keywords:** shoelaces, knots, impact

## Abstract

The accidental untying of a shoelace while walking often occurs without warning. In this paper, we discuss the series of events that lead to a shoelace knot becoming untied. First, the repeated impact of the shoe on the floor during walking serves to loosen the knot. Then, the whipping motions of the free ends of the laces caused by the leg swing produce slipping of the laces. This leads to eventual runaway untangling of the knot. As demonstrated using slow-motion video footage and a series of experiments, the failure of the knot happens in a matter of seconds, often without warning, and is catastrophic. The controlled experiments showed that increasing inertial effects of the swinging laces leads to increased rate of knot untying, that the directions of the impact and swing influence the rate of failure, and that the knot structure has a profound influence on a knot's tendency to untie under cyclic impact loading.

## 1. Introduction and motivation

**Figure 1.**
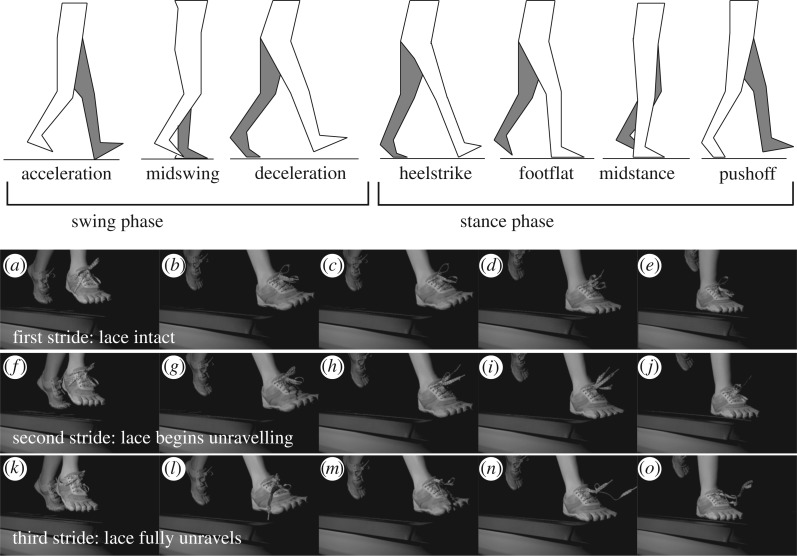
Illustration of the stages of the human gait and images from high-speed camera observation of shoelace knot failure. The three strides shown in the images come after several minutes of running on the treadmill. Images in the same column show the same stage in the stride. Each row of images corresponds to a single stride and the duration of each stride is approximately 1 s.

While most people have experienced accidental untying of their shoelaces, little is known and even less is documented about the physical mechanisms responsible for this ubiquitous annoyance. A popular 2005 TED Talk by Terry Moore on strategies for tying shoelaces to minimize knot failure, while helpful in suggesting a knot-tying heuristic, does not explain the source of the failure. Fortunately, some hints towards the nature of the shoelace failure mechanism can be understood anecdotally. We observed that a shoelace knot that often failed very quickly when walking (typically within 50 feet) did not fail when the leg was simply swung back and forth a similar number of cycles, that is, with no impact while sitting on a table. However, simply stomping the foot on the ground the same number of cycles also did not lead to untying. These observations suggest that the knot failure involves an interplay between the swing and stance phases (cf. figure 1) of the walking motion. Stated differently, failure does not occur without both the impact and swinging experienced during walking or running.

With the goal of observing shoelace knot failure, we examined slow-motion video footage of a runner on a treadmill (see electronic supplementary material, video S1) whose shoes were tied with what we call the weak knot (which is based on what is commonly termed the false, granny or granary knot). The resulting images of the knot failure were striking (figure 1). In particular, there appeared to be two time scales upon which untying took place: little change to the knot was observed for many strides until some untying began, after which the speed of untying was remarkable (often in less than two running strides).^1^ These observations informed both our hypothesized failure mechanism and experimental design.

We refer the reader to figure 1 for illustrations of the phases of a walking gate and appendix A (figure 14*a*–*g*) for a comparison of the structures of weak knots and strong knots. Our hypotheses for the failure of the knot are detailed in §3. We believe the failure is due to a combination of knot deformation associated with the impact of the shoe during the heel striking phase of the walking gait and the flapping motion of the shoelaces during the swinging phase of the walking gait. The deformation of the knot and the relative motion of the shoelace strands are moderated by the friction between the strands of shoelace in the knot centre to create the slow-fast (or ‘gradually-suddenly’) time scales. To formulate the hypothesis, we discuss scientific work on knots in §2. After the hypothesis has been presented, we then turn to an experimental validation of the proposed failure mechanism. The experiments are outlined in §4 and feature a custom-made impact test facility. Our work also presents some challenges to the computational mechanics community which we hope will inspire future work.

## 2. Background

**Figure 2.**
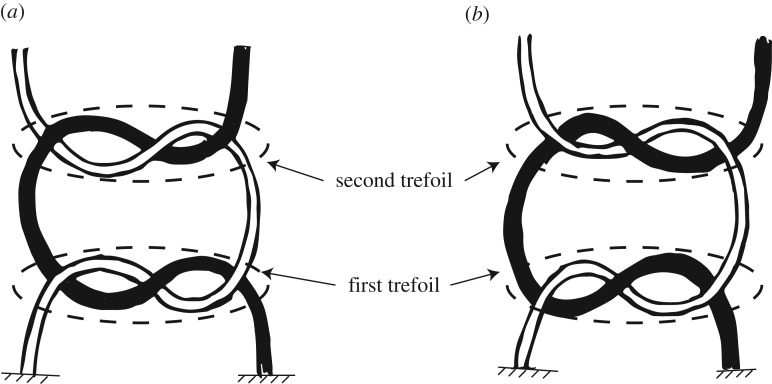
Illustration of both (*a*) the false knot and (*b*) the square knot, upon which the weak and strong knot are based, respectively. Each are composed of a series of stacked trefoils (a simple crossing of lace strands), with the ‘first trefoil’ referring to the one closest to the shoe—it is also the first tied. The difference between the knots lies in the handedness (the ordering of which lace crosses over which) of the ‘second trefoil’: that is, ‘left over right’ or ‘right over left.’ In the strong knot, the handedness of the second trefoil is different from the first.

In this paper, we deal primarily with two versions of the common shoelace bow-tie knot. The primary difference between the versions of this knot has to do with their component parts: a first trefoil, or lace crossing, tied close to the lacing and a second trefoil tied after the first (cf. figure 2 for an illustration of these trefoils). The strong version of this knot is based on the square knot: two trefoil knots of opposite handedness are stacked with the free ends tucked into the knot centre, thereby creating loops (cf. figure 14*h*,*i* in appendix A) for an illustration of the relationship between the two). The weak version of the knot, as mentioned previously, is based on what is commonly known as the granny or false knot. In contrast with the strong version, the two trefoils have the same handedness, causing the knot to twist instead of laying flat when tightened. These differences are illustrated in appendix A (figure 14*a*–*g*) and we encourage the reader to experiment with their own shoelaces. While the inferior performance of the weak version is well known in both common knot lore and surgical knot literature [1–4], we have yet to find a proposed structural characteristic to explain the inferior behaviour. Likewise, we have not found a study explaining the failure mechanism for a shoelace knot.

Scientific work on knots can be roughly divided into three major categories: mathematical study of knot topology, rod-based models for physical knots, and experimental investigations of surgical suture knots. The first category pertains to mathematical knot theory where invariants for knots are sought and studied (see [5,6] and references therein). The studies in this field allow one to classify and distinguish types of knots and determine invariants of a particular knot topology (such as trefoil (or simple) knots, cinquefoil (or double) knots, and figure eight knots) that cannot be changed without cutting the strands of the knot. In the second category, studies in the mechanics of physical knots seek to use an elastic rod model to determine the deformation of the strands of a knot [7,8]. Owing to the interest in simulating and animating strands of hair, ropes and sutures in computer graphics, progress in this area has been rapid in the past decade (cf. [4,9]). However, simulating the dynamics of the shoelace knot under conditions experienced during walking remains a challenging goal. An appreciation for the technical challenges can be gained by examining recent studies such as [8,10] on the mechanics of rods having tangled self-contacting strands. Adding to the technical challenges, several temporal scales must be accommodated in order to examine the failure of a shoelace knot: the short duration of the impact of the shoe with the ground during the heel strike and the much longer duration of the motion of the free ends of the lace. Further, several length scales must also be considered: the small zones dominated by friction and deformation of the shoelaces and the much larger segments of flapping lace.

The challenges of numerically modelling shoelaces led us to focus our efforts on an experimental examination of knot failure, and it is our hope that such experiments will inform future modelling work. Our work was aided by the large literature on experimental characterizations of knot strength and failure in surgical sutures. Unlike knot topology, which deals with arbitrarily self-tangled curves, the suture knots that are examined are typically simple combinations of different trefoils [11,12] that are similar to shoelace knots. Testing conducted in these studies features quasi-static and cyclic tests with no inertial effects [1,13–15]. Such tests typically lead to physical breaking rather than untying of the knot. Even if untying is accounted for, it is often secondarily commented on, and the concentration on breaking failures means that significant non-elastic effects have been experienced by the strands up until failure [13,16]. Significant plastic deformation is not a part of the average shoelace knot's untying, limiting the applicability of these studies to the problem at hand. However, the procedures and protocols gleaned from the literature on surgical sutures were invaluable to us when designing our experiment.

## 3. Hypothesized knot failure mechanism

**Figure 3.**
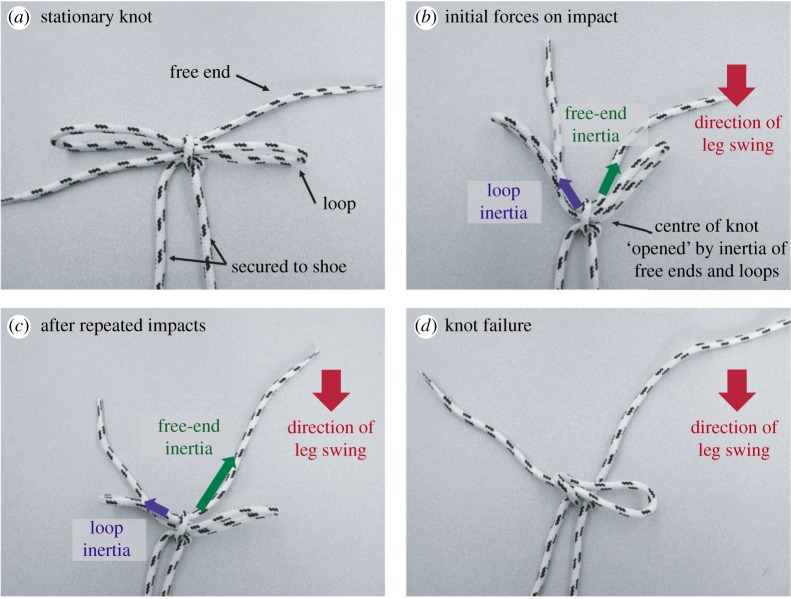
Static representation of hypothesized stages of knot failure. (*a*) Terminology for various parts of the knot. (*b*) As the leg is swung backwards to impact the ground, the inertia of the free ends and loops pull open the centre of the knot. If the free end and loops are approximately the same length, these forces will be comparable. If the knot centre is tightened, frictional forces will somewhat ameliorate the inertial force imbalance. (*c*) Repeated impact causes the centre of the knot to incrementally loosen which reduces frictional effects and magnifies the effects of the inertial imbalance between the free ends and loops. Additionally, the impact excitation causes slight pull through of the free end. This increases the inertial effects of the free end, enabling further free-end pull through. (*d*) Eventually, the inertial effect of the free end is sufficiently large that the knot fails suddenly and catastrophically when the free end is completely pulled through the centre of the knot, resulting in complete failure of the second trefoil. (Online version is colour.)

We developed a hypothesis for knot failure based on the aforementioned high-speed observations and additional normal-speed observations of knots in both normal operation (i.e. on a shoe, walking or running) and artificial operation (i.e. swinging a shoe at the edge of a table in order to isolate inertial effects, or stamping of a foot in place to isolate impulsive effects). Our hypothesis was further refined by data from the testing of knots mounted on the device described in §4. The hypothesized mechanism is as follows:

(i) as the leg is swung forward, and then slightly backwards to impact the ground, the loops and free ends of the shoelaces (figure 3*b*) are all pulled forward (with respect to the knot centre) by their inertia. The relative motion causes an opening of the knot—that is, a widening of the centre space separating the two trefoils. The centre space is where the free ends were pulled back through the knot to create the loops (cf. figure 14*h*,*i* in appendix A);
(ii) the impact force of the shoe during the striking of the heel is transmitted to the knot by the tongue of the shoe and the eyelets. As a result, the centre of the knot deforms;
(iii) the opening of the knot, and the concomitant reduction in friction forces, facilitates relative motion of the free ends with respect to the knot centre. In other words, with the centre of the knot pulled apart, relative axial motion between the free end and the knot centre becomes possible, and it is easier for the free end to slide out of the knot centre. The tension force pulling a free end through the centre is due to the imbalance of the inertia of the free end (the same inertia that helps to pull the knot apart), the inertia of the corresponding loop (to which the free end attaches through the knot), and the frictional forces at the centre of the knot;
(iv) the repeated impacts perturb the knot such that the free end is incrementally pulled through the knot. This happens slowly at first. But as the knot is repeatedly pulled apart and more length is fed through the knot centre to the free end, the inertial forces pulling the free end through the knot are increasingly magnified, while the competing inertia forces of the loop diminish as the loop size decreases (figure 3*c*); and
(v) eventually, the free end is sufficiently long that in one or two strides (impact cycles), the inertial force associated with the free end completely overpowers the loop's inertial force, causing run-away knot failure as the free end pulls completely through the knot centre. This last event signifies total failure of the second trefoil (figure 3*d*).

It should be noted that this hypothesized mechanism explains the characteristic speed at which catastrophic knot failure occurs. We have framed the hypothesis assuming that the inertia of the free end dominates that of the loop (an assumption that matched the vast majority of observed failures). The corresponding hypothesis when the opposite situation arises is readily formulated. In this case, the loop gets larger at the expense of the free end and the failure mechanism is similar to the case discussed in detail above. However, we believe this is rarely observed because the loops are necessarily constrained in the orientations they can take, and do not undergo the same range of motion/acceleration as the free ends (which quite literally ‘whip’ back and forth).

We suspect, but have not been able to prove, that the difference between the weak knot and the strong knot failure rates lies in the twist of the weak knot. The strong knot can be tightened, yet remain planar. However, to tighten the weak knot, the structure must twist completely. For the knot to fail, the deformation of the knot centre noted in (ii) should contribute to the opening (rather than tightening) of the knot centre, but it has proven elusive to measure the deformation of the knot centre under a heel strike.

## 4. Experiments

### (a) Preliminary testing: *in situ* high speed failure observation and force measurement

To inform controlled laboratory experiments and to hone suspected failure hypotheses, initial knot failure experiments were performed *in situ* on shoes during running and walking. The first of these *in situ* experiments observed knot failure using a high-speed camera (Vision Research Phantom Miro M110, 1280 × 800 pixels, 900 fps). A volunteer runner was instructed to tie her shoes using the weak version of the standard shoe knot. The volunteer then ran on a treadmill (cf. figure 1; electronic supplementary material, video S1) until acute knot failure was observed (defined as failure of the second trefoil, more commonly understood as loop untying). The observed failure occurred rapidly and without warning, with footage confirming that full failure occurred within one to two strides of failure initiation. A single failure mode was observed where a free shoelace end pulled through the knot, thus reducing it to a single trefoil. The same volunteer was additionally instructed to tie her shoelaces using the strong version of the standard shoe knot—with approximately the same initial tightening as the weak knot—and no acute failure (that is, complete untying) was observed during the testing period.

To better understand the dynamic conditions surrounding failure, we measured the accelerations acting on the knot during the motions of walking and running. A LORD Microstrain 10g wireless accelerometer was attached to multiple types of shoes directly underneath the centre of the knot (a location which varied slightly depending on the shoe). Shoes included in the initial study were typical running shoes, hiking boots, casual sneakers and barefoot shoes. With a metronome to help her maintain a constant cadence, a volunteer was asked to both walk and run on a straight, level surface, and the accelerometer's time history was recorded. This time history contained measurements resulting from inertial and impact sources.^2^ The impact sources produced accelerations which were large and impulsive. For the walking tests, the impact magnitude (or measured acceleration at the base of the knot trefoils) was typically on the order of 6–8*g*. Furthermore, analyses of testing results showed surprisingly similar magnitudes of acceleration regardless of shoe type (a schematic of the *in situ* mounting and time history is presented in appendix B). As we wished to study untying that occurs even in the least extreme cases (i.e. slow walking on a thick sole, as opposed to the extreme case of running on a thin sole), it was resolved to focus further testing on impulses corresponding to this regime. This information was used to calibrate the impact magnitude—chosen to be approximately 7*g*—for the controlled testing described in the following section.

### (b) Controlled testing: cyclic impact

**Figure 4.**
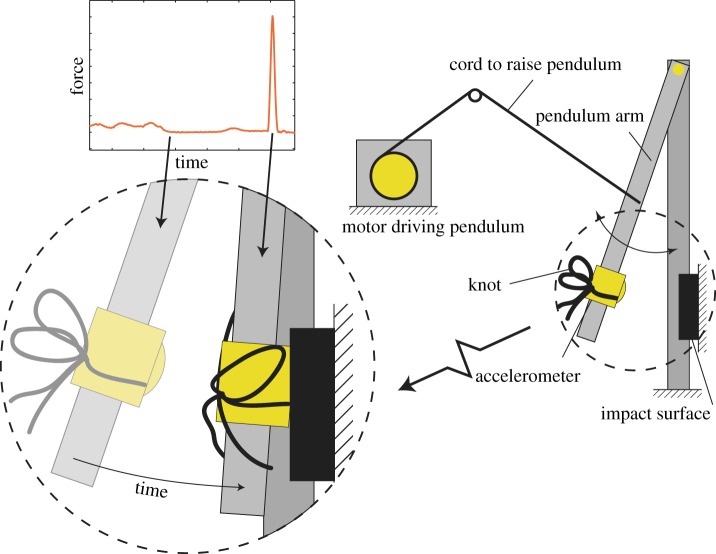
Illustration of the experiment used to study the effects of impact and lace dynamics on a knot. The knot is mounted to a pendulum arm (approximately 20 cm in length) that is released from rest at a prescribed angle of inclination and impacts a surface. The angle was chosen to be approximately 43^°^. This choice of angle, combined with a tuning of the impact surface, produced an impact deceleration of ≈7*g*. (Online version in colour.)

**Figure 5.**
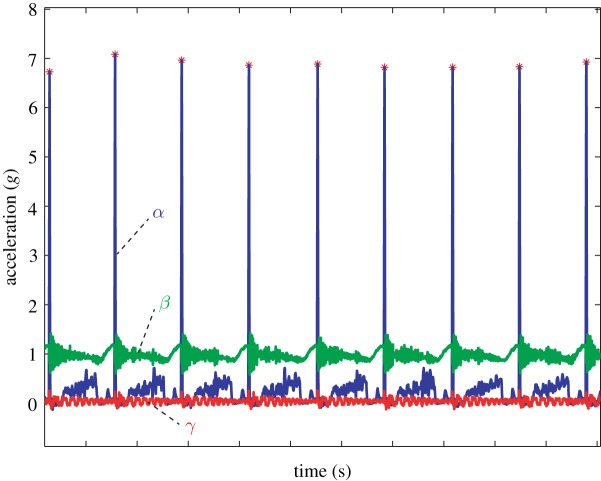
Representative acceleration time histories for the impact testing from the pendulum apparatus shown in figure 4. The blue curve (labelled *α*) represents the accelerations experienced by the knot in the impact direction. The green curve (labelled *β*) and red curve (labelled *γ*) represent off-axis accelerations in the vertical and lateral directions, respectively. (Online version in colour.)

We fabricated an experimental set-up to isolate the effects of impact magnitude, impact direction, and free-end inertial force magnitude on knot failure. Illustrated in figure 4, an actuated pendulum was constructed to generate a primarily unidirectional impact force acting on the knot centre (see electronic supplementary material, video S2). The pendulum was dropped from an adjustable height to control the impact magnitude, and the mounting orientation of the knot determined the effective impulse direction. An example of the time history is shown in figure 5. The impact surface was tuned to produce minimal off-axis excitation and provide an impulse profile characterized by a single 7*g* peak similar to those observed in the accelerometer data mentioned previously (cf. appendix B). The pendulum was tuned such that mean off-axis accelerations were, at most, 10% of the mean peak on-axis acceleration values. In most cases, they were less than 5%.

Except when otherwise noted, the strong knot was used for all tests. A rigorous knot tying procedure and a template were developed to ensure uniformity of knots between tests. Templates were used to manage knot geometry (size of free ends and loops) and tightening of the knots. For the latter, knot tightening was standardized by hanging weights from knot loops. Initially, the knots were tied so that the loops and the free ends were of equal length (8.26 cm). The free-end length was defined as the distance of the lace end relative to the knot centre and the loop length was defined as the distance of the middle of the outstretched loop (i.e. half of its arc length/chord length) relative to the knot centre (cf. appendix A (figure 14*j*)). All knots studied were tied with identical store-bought black (unwaxed) dress laces with a cross-sectional diameter of 2 mm. An attempt was made to characterize the bending stiffness of the laces, but values determined via an optical and force-based method were on the order of the error of the method (10^−6^ Nm^2^). The exceptionally small value of the bending stiffness led us to conclude that it is of negligible importance relative to frictional terms.

All experiments measured the rate of knot failure. The length of the free end was measured at the beginning of the experiment and either after knot failure, if the failure occurred within a trial timeout of 15 min, or at the end of the trial. This was then divided by the total number of impact cycles to calculate an average slip rate (average change in the length of the free end per impact cycle).

**Figure 6.**
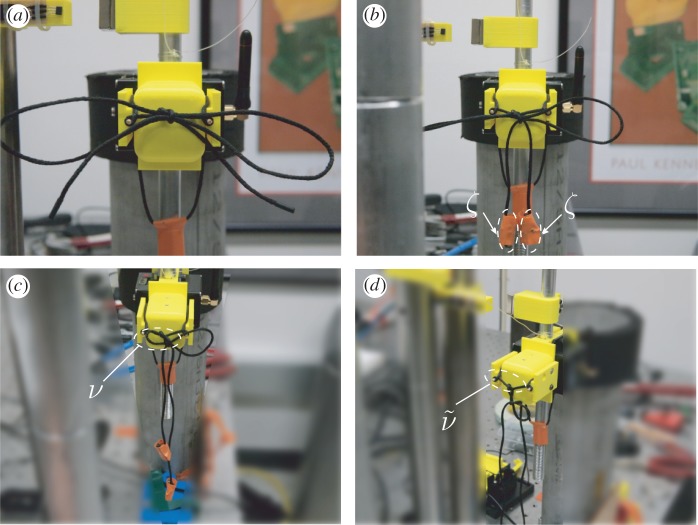
Example of a strong knot: (*a*) mounted on pendulum; (*b*) with attached free-end weights (*ζ*) to simulate added inertia effects; (*c*) partially failed (diminished loop indicated by *ν*); and (*d*) completely failed (absent loop indicated by $\tilde{\nu }$). It should be noted that the knot shown in (*c*) has a diminished loop size and extended free ends compared to the knot displayed in (*b*). (Online version in colour.)

In our preliminary testing, we observed that knot failures were almost exclusively ‘pull through’ in nature, suggesting that the inertia of the free ends dominated that of the loops. To isolate the effect of free-end inertial force asymmetry on knot failure, masses of various sizes were added to the free ends to magnify their inertial effect. The values of these masses were chosen heuristically so that during the transition from the lowest value to the highest value a corresponding change from limited knot failure to frequent knot failure was observed. End masses of 1 g, 2 g and 3 g represented 7.4, 14.8 and 22 times the initial mass of the free end and 3.7, 7.4 and 11.1 times the initial mass of the loop, respectively, were added to the free ends of the shoelaces. The effect of different masses on the average slip rate was measured (figure 6).

**Figure 7.**
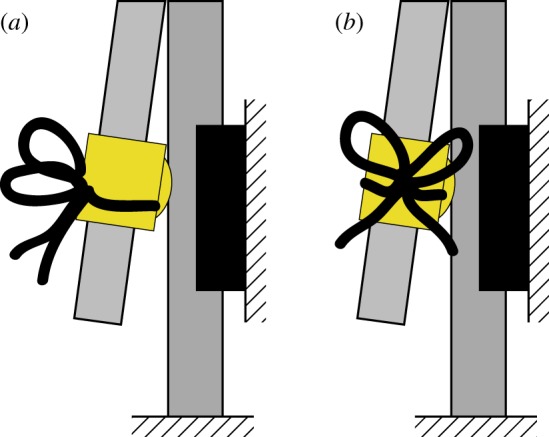
Schematic of impact orientations of the knot, relative to the impulsive loading direction: (*a*) ‘rear impact’ and (*b*) ‘side impact’. (Online version in colour.)

**Figure 8.**
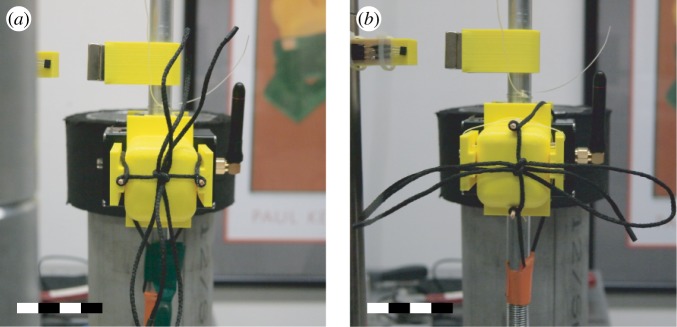
Comparison of the orientations of the weak knot on the pendulum apparatus: (*a*) in a horizontally mounted configuration and (*b*) a vertically mounted configuration. In both figures, the scale bar has a length of 4 cm. (Online version in colour.)

The effect of impact direction on failure rate was investigated by mounting the knot on the pendulum in different orientations. We concentrated on two orientations: a ‘rear impact’ where the plane in which the two trefoils lie is normal to the impacting direction and a ‘side impact’ where that same plane is parallel to the impacting direction. We can conceptually move from a ‘rear impact’ to a ‘side impact’ by rotating the knot 90^°^ about the axis of the gravitational force (cf. figure 7). Additionally, the failure frequency of the strong knot was compared with the weak knot. As the weak knot does not orient itself along the same axis as the strong knot, two mounting orientations of the weak knot were tested (cf. figure 8).

## 5. Results and discussion

**Figure 9.**
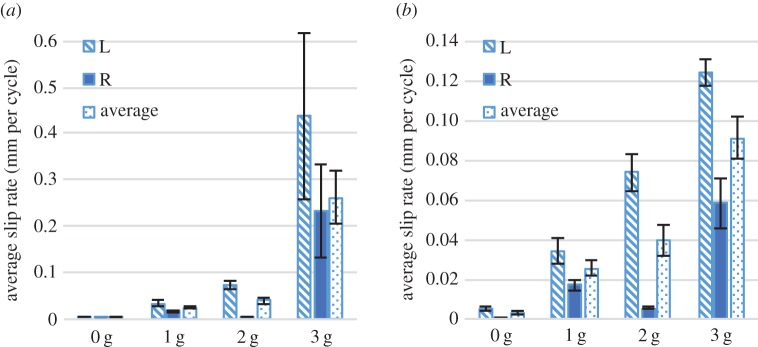
(*a*) Average rate of change of the length of the free end for rear-impacted knots with different free-end masses. (*b*) Average rate of change of the length of the free end for rear-impacted knots with different free-end masses, excluding specimens that failed completely in the 3 g sample. For both subfigures, the label ‘L’ refers to the left free end, the label ‘R’ to the right free end, and the label ‘average’ to the mean of the left and right free ends. Error bars show standard error. (Online version in colour.)

We found two regimes of knot failure: gradual loosening and acute failure. To characterize these regimes, we define the average slip rate for each test as the average change in length of the free end per impact cycle (calculated by dividing the net change of the length of the free end during testing by the total number of impact cycles). Total or acute failure was defined as the complete unravelling of the second trefoil. Figure 9*a* shows the average slip rate for the rear impact test, with 0, 1, 2 and 3 g free-end weights. The labels ‘L’, ‘R’ and ‘Average’ correspond to the change in length of the left free end, right free end and the average of the two, respectively.

Only tests conducted with 3 g weights experienced acute knot failure, which occurred for approximately half of the 3 g tests (53% or 8/15 tests). It is clear that tests using 3 g free-end weights entered a regime of force asymmetry between the loop and free end that caused significantly more rapid failure. However, we observed that acute failure was runaway in nature: the majority of the free-end length change (and thus failure) occurred within a relatively short number of impacts for all tests and amounts of free-end weight. This behaviour is consistent with observations from high-speed video and is evident when examining the isolated slip rates of the 3 g tests that did not fail (figure 9*b*). If instances of runaway failure are excluded, the increase in average slip rate increases approximately linearly with free-end weight (i.e. loop/free end inertial asymmetry), whereas comparing the average slip rate of all 3 g trials shows a more than fourfold increase in slip rate that is heavily weighted by runaway failure tests. The rear impact tests showed a significant bias towards the failure of the left lace which was probably due to the initial knot structure.

**Figure 10.**
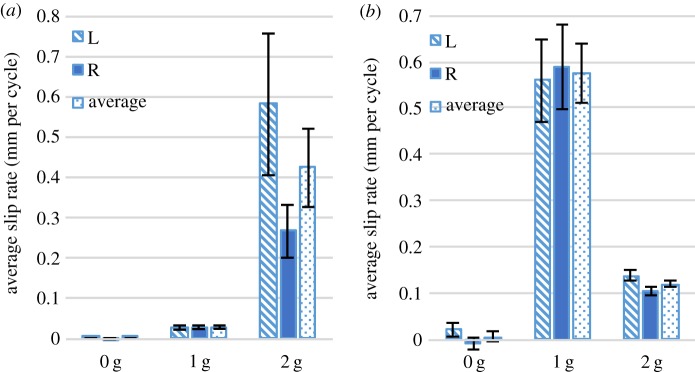
(*a*) Average free-end slip rate of the free end for side-impacted knots with different free-end masses. (*b*) Average free-end slip rate for side-impacted knots with different free-end masses, excluding specimens that failed completely in the 2 g free-end weight sample. For both subfigures, the label ‘L’ refers to the left free end, the label ‘R’ to the right free end, and the label ‘average’ to the mean of the left and right free ends. Error bars show standard error. (Online version in colour.)

Figure 10*a* shows the average free-end slip rate for the side impact test, with 0, 1 and 2 g free-end weights. Only tests conducted with 2 g weights experienced full knot failure. As with the rear impact tests, side impact tests exhibited runaway failure. Therefore, figure 10*b* compares the average slip rates for the different free-end weights, excluding the 2 g tests that experienced full runaway failure. Unlike the rear impact tests, the side impact slip rates for non-failed knots did not monotonically increase with increasing free-end weight (increasing inertial force asymmetry). The reason for this behaviour is not fully understood and requires further investigation. Interestingly, biasing of failure between the left-free end and right-free end was not observed in the side impact tests as it was in the rear impact tests.

**Figure 11.**
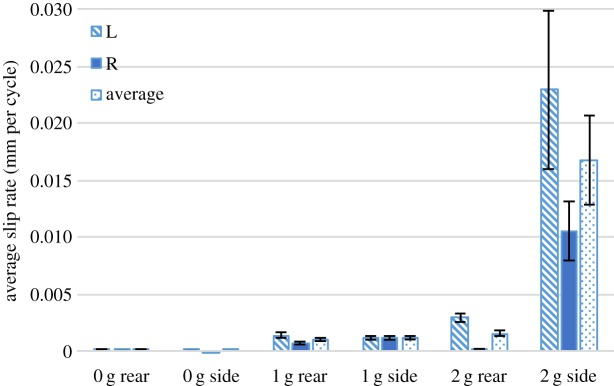
Average free-end slip rate for side-impacted knots compared with rear-impacted knots with different free-end masses. The label ‘L’ refers to the left free end, the label ‘R’ to the right free end, and the label ‘average’ to the mean of the left and right free ends. Error bars show standard error. (Online version in colour.)

**Figure 12.**
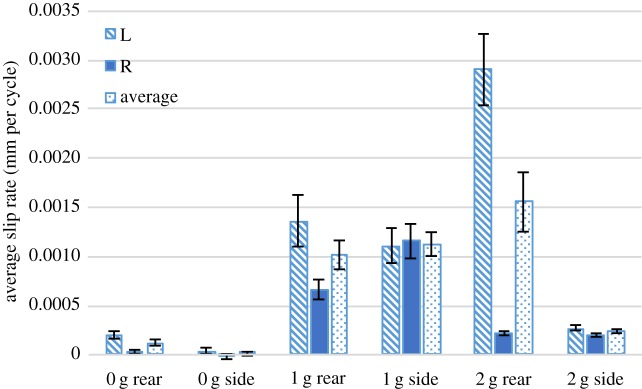
Average free-end slip rate for side-impacted knots compared with rear-impacted knots with different free-end masses excluding specimens that failed completely. The label ‘L’ refers to the left free end, the label ‘R’ to the right free end, and the label ‘average’ to the mean of the left and right free ends. Error bars show standard error. (Online version in colour.)

Figures 11 and 12 show comparisons of the average free-end slip rates of side- and rear-impacted knots with differing free-end weights. In both the side and rear impacts, an inertial mass threshold was observed where total failure begins to occur within the allotted test time of 15 min. While no significant difference was seen between the rear and side impact slip rates for different weights, it should be noted that knots impacted from the side experienced total failure at lower amounts of the free-end weights than the laces subjected to a rear impact (40% of side-impacted knots failed with 2 g free-end weights, while no rear-impacted knots failed with 2 g free-end weights). While this does suggest some dependence of the failure on direction of impact, more experimentation is necessary to characterize this effect.

**Figure 13.**
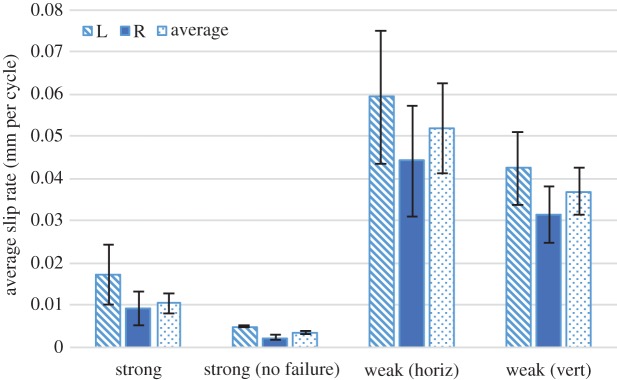
Average free-end slip rate for the rear-impacted weak version of the knot compared with the rear-impacted strong version of the knot, with a free-end weight of 3 g. It should be noted that because of the inherent chirality of the weak knot, two orientations of this knot were tested. The pair of orientations can be seen and compared in figure 8. The label ‘L’ refers to the left free end, the label ‘R’ to the right free end, and the label ‘average’ to the mean of the left and right free ends. Error bars show standard error. (Online version in colour.)

**Figure 14.**
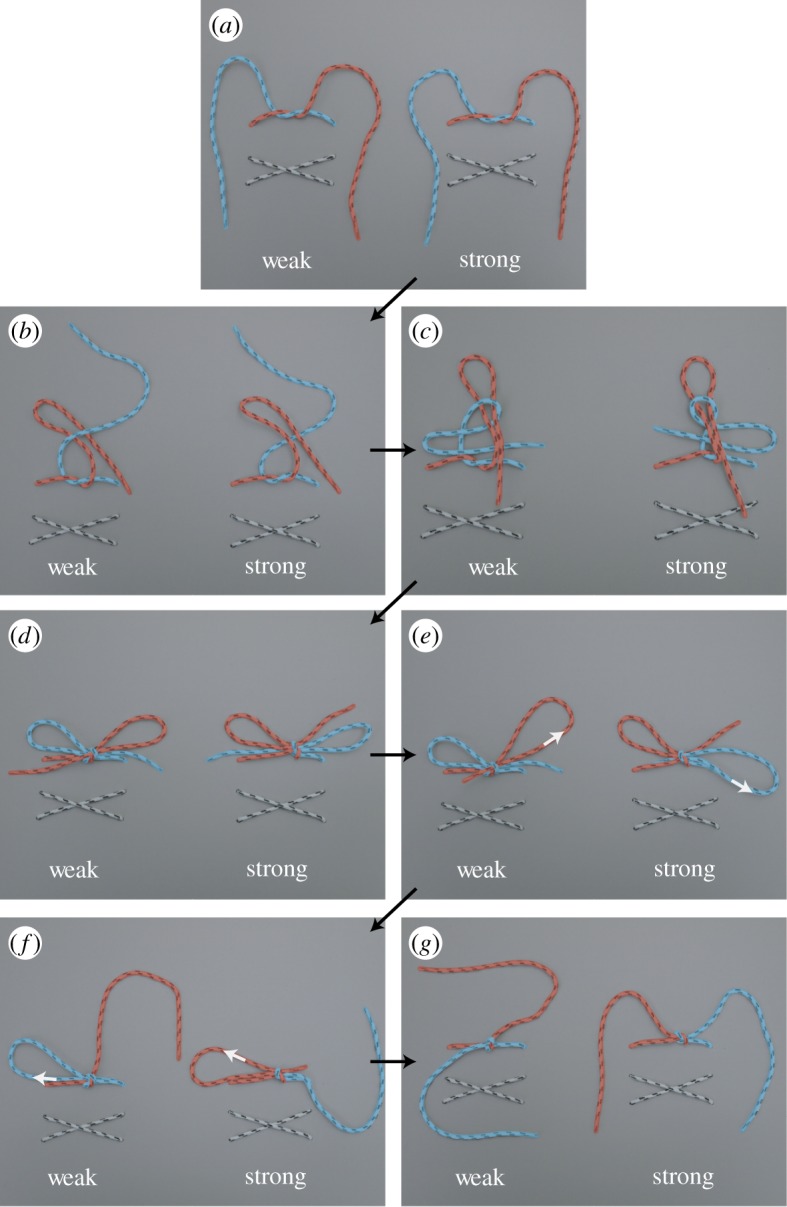

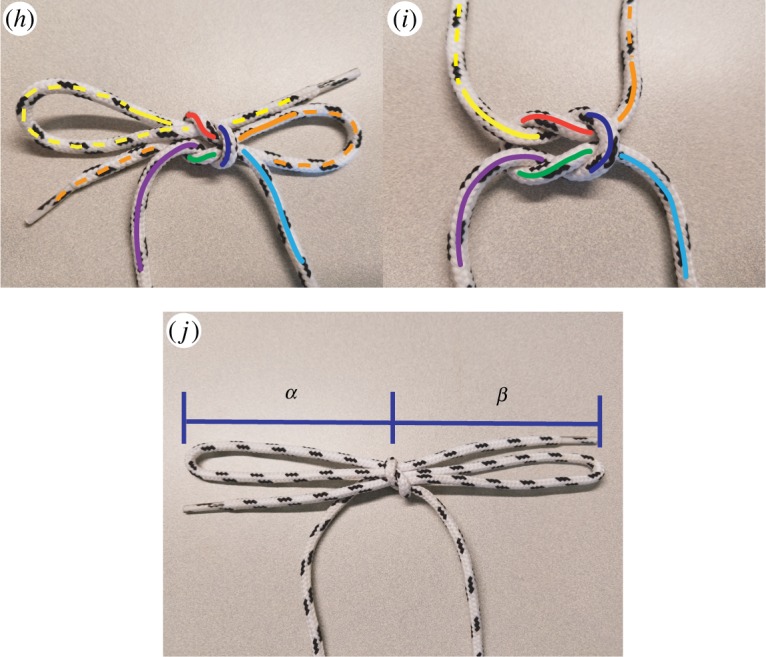
An overhead view of the tying of the weak knot (on the left of each image panel) and the tying of the strong knot (on the right of each image panel). Note, importantly, that the two knots only differ in the relative tying of the second trefoil to the first trefoil (cf. panels (*b*) and (*c*)). Further, after the loops are pulled out (indicated by the white arrows in panels (*e*) and (*f*)) the weak and strong knots become the prototypical false/granary/granny and square/reef knot (cf. panel (*g*)), respectively. Depictions of the (*h*) strong knot and (*i*) square knot illustrating their underlying structure. Colour coding shows equivalent knot sections and demonstrates that the strong knot is equivalent to the square knot with its free ends (yellow and orange) tucked into the knot centre to create loops. Subfigure (*j*) shows the relative dimensions of a knot tied according to the template. The free-end length (*α*) is equal to half of the loop chord length (*β*). (Online version in colour.)

As expected, the weak knot failed much faster than the strong knot. It is commonly known in surgical knot practice and general knot lore that the disparity of load-carrying capabilities of the weak knot compared with the strong knot are significant. Figure 13 compares average free-end slip rate for both the strong and the weak knot with added 3 g free-end weights, impacted from the rear. The weak knot experienced a 100% failure rate in both mounting configurations and at much higher slip rates than the strong knot. Differences in slip rate between the two weak knot mounting orientations did not show significant differences.

## 6. Conclusion

High-speed video observation of *in situ* shoelace knot showed failure to be a sudden and catastrophic phenomenon. Observations point to a failure driven by the complex interplay between impact-induced deformation of the centre of the knot, dynamic swinging of the walking motion, and the inertial forces of the laces and free ends of the knot. Preliminary experimental results showed that runaway failure and loosening can be linked to a mismatch between the inertial forces of the loop and the free ends that is decreasingly mediated by friction as the knot centre is loosened under cyclic impact. The increasing mismatch leads to an increase in slip rate and causes an accelerating failure. Our results also confirmed that the weak knot fails at higher slip rates and frequency than the strong knot. Further testing is necessary to more fully understand the effect of impact orientation on the weak knot versus the strong knot.

It was also shown that knot failure was runaway in nature, with the majority of the change in length associated with complete knot failure occurring in just a few impact cycles. The direction of the impact has an effect on failure rate, with side impacts on the strong knot entering a regime of full failure at a lower threshold of inertial asymmetry (within the allotted testing time of 15 min) than the rear-impacted knots. This is suspected to have implications for differences of failure rate between shoe types observed anecdotally, as the location and orientation of the knot on the shoe leads to a different direction of the resultant impact force.

Our work on the failure of the shoelace knot is far from exhaustive. For one, the influence of the shoelace material and surface finish was not investigated. In addition, the metric used to understand knot slip rate in our work (measurements of free-end length before and after set testing time or failure) is insufficient to elucidate finer details of knot failure progression from gradual loosening to a regime of fast failure. To do so, future tests should fail each knot, measuring free-end lengths at set intervals during testing. Such measurements will better distinguish the two regimes of knot failure we have identified (one regime where the inertial imbalance between free ends and loops is relatively stable, the other where the imbalance rapidly leads to runaway knot untying). We expect that such measurements and further testing will also lead to better understanding of the mechanical factors that cause the inferiority of the weak knot compared with the strong knot.

## Figures and Tables

**Figure 15.**
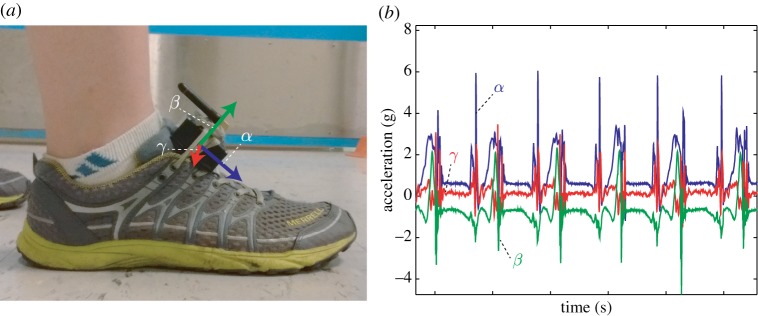
(*a*) Photograph of *in situ* testing of the accelerations experienced by a shoelace knot. Illustration of the recorded acceleration axes is given: the primary direction of impact lies in the plane formed by the blue arrow (labelled *α*) and the green arrow (labelled *β*). The red arrow (labelled *γ*) signifies the direction of off-axis accelerations. (*b*) Accelerations as functions of time in three orthogonal directions for the *in situ* testing of the accelerations experienced by a shoelace knot; *α*, *β*, *γ* as before. (Online version in colour.)

## Footnotes

1 The speed of the knot failure brings to mind a Hemingway line from *The Sun Also Rises* describing a character's descent into bankruptcy—which here we found to be an apropos description of knot failure—it happens ‘Two ways. Gradually, then suddenly.’

2 The accelerations experienced by the centre of the knot are not necessarily the same as those experienced by the loops or free ends. The loops and free ends will experience additional accelerations relative to the knot centre which we were unable to measure directly.

## Data accessibility

Experimental data can be accessed at https://doi.org/10.6078/D15K5K.

## Appendix A

See figure 14.

## Appendix B

See figure 15.
